# The Impact of Mitral Valvular Etiology on Left Atrial Functional Recovery After the Maze Procedure: A Comparison Between Mitral Stenosis, Mitral Regurgitation and Non-Mitral Valve Disease

**DOI:** 10.3390/jcm15051856

**Published:** 2026-02-28

**Authors:** Woo Sung Jang, Jung Uk Woo, Kyungsub Song

**Affiliations:** 1 Department of Thoracic and Cardiovascular Surgery, Keimyung University Dongsan Hospital, Keimyung University College of Medicine, Daegu 42601, Republic of Korea; whiteuri@dsmc.or.kr; 2 School of Medicine, Keimyung University College of Medicine, Daegu 42601, Republic of Korea; junguk1427@gmail.com

**Keywords:** atrial fibrillation, maze procedure, left atrial function, speckle-tracking echocardiography

## Abstract

**Background**: Although the concomitant Maze procedure successfully restores sinus rhythm in patients with valvular atrial fibrillation, it remains unclear whether electrical restoration translates into uniform functional recovery across different valvular etiologies. To address this issue, we compared the long-term left atrial (LA) mechanical recovery between patients with mitral stenosis (MS) and mitral regurgitation (MR) after the Maze procedure. **Methods**: This retrospective study included 211 patients who underwent the Maze procedure concomitant with valvular surgery and maintained sinus rhythm after 1 year. Patients were stratified into three groups, namely MS (*n* = 51), MR (*n* = 98), and non-mitral (*n* = 62) serving as a reference. LA function was evaluated using speckle-tracking echocardiography at baseline, immediately postoperatively, and at 1 year. Primary outcomes were changes in LA reservoir (LASr), LA conduit (LAScd), and LA contractile (LASct) strains. **Results**: At 1-year follow-up, the non-mitral reference group exhibited the best LA function, followed by the MR group, whereas the MS group showed the most impaired values (*p* < 0.001). Analysis of functional recovery revealed a mechanistic divergence, i.e., although the improvement in passive stiffness (LAScd) was comparable between the MS and MR groups (*p* = 0.42), the recovery of active contractile strain (LASct) was significantly superior in the MR group compared to the MS group (*p* < 0.05). The MS group failed to regain effective atrial contraction despite successful rhythm control. **Conclusions**: Although the Maze procedure successfully restored sinus rhythm, functional recovery varied significantly by etiology. The superior recovery in patients with MR was driven by the restoration of active atrial contraction, whereas patients with MS exhibited persistent mechanical dysfunction attributed to irreversible myocardial structural remodeling, despite similar improvements in compliance. Therefore, electrical success does not guarantee functional success, particularly in patients with MS.

## 1. Introduction

Atrial fibrillation (AF) is a common comorbidity in patients with mitral valvular disease, which significantly increases the risk of stroke and heart failure along with adversely affecting long-term survival [1,2]. Consequently, the Maze procedure, which is performed concomitantly with mitral valve surgery, has become the standard of care for rhythm control. With advancements in surgical techniques, the restoration of normal sinus rhythm (NSR) is now achievable in approximately 78–84% of patients postoperatively [3].

Although earlier studies primarily focused on the successful conversion to NSR on electrocardiography, recent attention has shifted toward the recovery of actual left atrial (LA) mechanical function [4,5]. In particular, LA contractile (LASct) strain, evaluated by echocardiography, has emerged as a critical parameter for determining LA function. It serves as a valuable indicator of LA reverse remodeling and a predictor of prognosis after the restoration of NSR [6,7]. Clinically, the persistence of LA mechanical dysfunction is not merely a hemodynamic parameter; it is closely linked to atrial stasis, increasing the risk of thromboembolism despite sinus rhythm [5], and compromises ventricular filling, potentially contributing to heart failure with preserved ejection fraction (HFpEF) [6,8].

Nevertheless, the hemodynamic loads imposed on the left atrium differ fundamentally according to the etiology of the valvular disease [9,10,11]. Essentially, mitral stenosis (MS) imposes chronic pressure overload on LA, whereas mitral regurgitation (MR) is characterized by volume overload on LA. These distinct hemodynamic burdens suggest that the patterns and extent of LA reverse remodeling diverge significantly between the two pathologies, even in the setting of maintained NSR after the Maze procedure. In this context, to accurately isolate the specific impact of mitral pathology from the general effects of cardiac surgery and ablation, a comparison with a reference group free from mitral valvular hemodynamic burden is essential.

However, there are currently limited comparative data on LA functional recovery—specifically regarding LASct—between patients with MS and MR after the Maze procedure. Therefore, this study was conducted to examine the characteristics of LA reverse remodeling in patients with MS and MR who maintained NSR after surgery. We hypothesized that despite successful electrical restoration, functional recovery would diverge according to etiology, with MS patients exhibiting impaired recovery compared to both MR patients and a non-mitral reference group.

## 2. Materials and Methods

### 2.1. Ethical Statement

The study protocol was approved by the Institutional Review Board of Dongsan Medical Center (IRB No. 2025-11-062; approved 25 November 2025). The requirement for written informed consent was waived by the Medical Ethics Committee of Dongsan Medical Center, Keimyung University School of Medicine, due to the retrospective nature of the study. All procedures were conducted according to relevant guidelines and regulations.

### 2.2. Study Population

**Figure 1:**
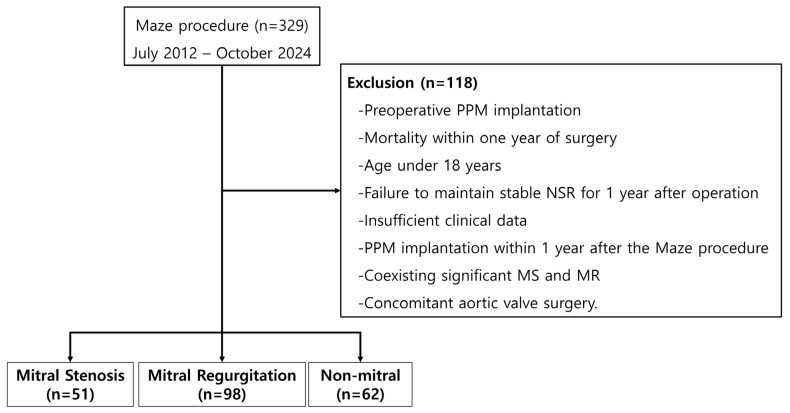
Study flow diagram.

We retrospectively examined the data of 329 patients who underwent the Maze procedure at our institution between July 2012 and October 2024 (Figure 1). To determine the impact of recovered LA mechanical function, we analyzed the clinical outcomes based on transthoracic echocardiography (TTE) parameters measured at 1-year follow-up.

To eliminate the downstream hemodynamic impact of aortic valve pathology on LV and LA function [8,12], we excluded patients with significant aortic regurgitation or stenosis based on the following criteria: (1) preoperative permanent pacemaker (PPM) implantation, (2) mortality within 1 year of surgery, (3) age < 18 years, (4) failure to maintain stable NSR at the 1-year follow-up, (5) PPM implantation within 1 year after the Maze procedure, (6) insufficient clinical or follow-up data, (7) coexisting significant MS and MR, and (8) concomitant aortic valve surgery.

Patients were categorized into the following three groups: MS, comprising those who underwent surgery for MS; MR, comprising those who underwent surgery for MR; and non-mitral, comprising patients who underwent the Maze procedure concomitantly with surgery for non-mitral valve diseases.

### 2.3. Echocardiography and Rhythm Follow-Up

Echocardiographic analysis was performed by TTE using commercially available ultrasound systems (Vivid E95, GE Vingmed Ultrasound AS, Horten, Norway; or Acuson SC 2000, Siemens Healthineers, Erlangen, Germany). Standard echocardiographic parameters, including left ventricular ejection fraction (LVEF), left atrial volume index (LAVI), and left ventricular end-diastolic dimension (LVEDD), were measured according to the guidelines of the American Society of Echocardiography [13].

LASct strain was evaluated using two-dimensional speckle-tracking echocardiography from the apical 4- and 2-chamber views. Electrocardiographic R-R gating was employed, with the onset of the QRS complex serving as the zero-strain reference point. The region of interest was manually adjusted to ensure optimal tracking of the LA endocardium. LASct, which represents the active booster pump function of the left atrium, was defined as the absolute change in strain during late diastole. To ensure measurement consistency and minimize inter-observer variability, all strain analyses were strictly performed by a single experienced echocardiographer who was completely blinded to the patients’ specific clinical groups and surgical outcomes.

In accordance with standard echocardiographic conventions, LASct is expressed as a negative value to reflect active myocardial shortening during atrial contraction; therefore, a more negative value denotes a stronger atrial contraction.

Immediate postoperative echocardiography was performed before discharge, typically on postoperative day 5 to 7, after the patient’s hemodynamic status had stabilized. Rhythm follow-up standard 12-lead ECGs were recorded daily during hospitalization. After discharge, outpatient ECGs were obtained at 1, 3, and 6 months postoperatively and every 3–4 months thereafter. To confirm rhythm status, 24 h Holter monitoring was performed at the 1-year follow-up visit.

### 2.4. Definitions

Maintenance of NSR at 1 year was defined as NSR confirmed by 24 h Holter monitoring or sustained NSR observed on consecutive outpatient follow-up ECGs. Regarding preoperative AF characteristics, “fine f-wave” was defined as fibrillatory waves (f-waves) with an amplitude of <0.05 mV (<0.5 mm) in lead V1, whereas “coarse” AF was defined as an amplitude of ≥0.05 mV. major adverse cardiac Events (MACE) was defined as a composite of cardiac death, nonfatal stroke, and hospitalization for heart failure.

### 2.5. Operative Techniques of Maze Surgery

A modified Cox Maze procedure was performed using cryoablation under antegrade or retrograde cardioplegia, according to established principles [14]. The LA lesion set comprised pulmonary vein isolation and a mitral isthmus line. The right atrial (RA) lesion set included an intercaval line, a “T” lesion extending to the tricuspid annulus, and a line involving the RA appendage. LA appendage obliteration was mandatory for patients with a history of stroke or intracardiac thrombus and was otherwise performed at the surgeon’s discretion.

### 2.6. Postoperative Anticoagulation Management

All patients received warfarin for 3 months postoperatively, with a target international normalized ratio (INR) of 2.0–3.0. Following this period, patients with mechanical prosthetic valves continued indefinite anticoagulation. For the remaining patients, antithrombotic therapy was either continued or switched to antiplatelet agents based on rhythm status and CHADS2 score.

### 2.7. Study Outcomes

The primary outcome was the serial change in LA systolic function, which was evaluated using LASct, from the immediate postoperative period to the 1-year follow-up in patients who maintained NSR. The secondary outcomes were MACE, defined as a composite of stroke (cerebral infarction), readmission for heart failure (HF), and cardiac death.

### 2.8. Statistical Analysis

Continuous variables are represented as the mean ± standard deviation, and categorical variables are expressed as frequencies and percentages. Comparisons of continuous variables among the three groups were performed using one-way analysis of variance. For post hoc pairwise comparisons, Welch’s *t*-test was used to account for potential heterogeneity of variances. Categorical variables were compared using Pearson’s chi-square test or Fisher’s exact test, as appropriate. To account for multiple pairwise comparisons, a Bonferroni correction was applied to the significance level.

Serial changes in echocardiographic parameters measured across three time points (preoperative, immediate postoperative, and 1-year follow-up, i.e., LAVI, LVEDD, and EF) were evaluated using a Repeated Measures Analysis of Variance (RM ANOVA) to account for within-subject correlations. For variables measured at only two specific time points (i.e., postoperative and 1-year LASct), temporal changes were analyzed using paired *t*-tests. Independent predictors of 1-year LASct and its absolute change were identified using multivariable linear regression analysis. Variables that showed statistical significance (*p* < 0.05) in the initial univariate analysis were selected as covariates for the final multivariable model.

All statistical tests were two-sided and conducted using Python (version 3.10). Due to the retrospective design of the study, a formal a priori sample size calculation was not performed. However, the highly significant differences (*p* < 0.001) and robust confidence intervals observed in the primary outcome (LASct) indicate that the available sample size provided adequate statistical power to detect meaningful clinical differences. A *p* value of < 0.05 was considered statistically significant.

## 3. Results

### 3.1. Baseline Characteristics

A total of 211 patients were included in the final analysis, who were categorized into three groups, namely MS (*n* = 51), MR (*n* = 98), and non-mitral (*n* = 62). Table 1 summarizes the baseline characteristics of the study population.

Mean age showed no significant differences among the three groups (*p* = 0.07). However, the proportion of male patients was significantly higher in the non-mitral group (60.3%) than in the MS group (31.4%) (*p* = 0.004 for MR vs. non-mitral). Regarding comorbidities, the prevalence of a history of stroke was significantly higher in the non-mitral group (25.4%) than in the MR group (8.1%, *p* = 0.005). Heart failure was most prevalent in the MR group (39.4%), exhibiting a significant difference compared with that in the non-mitral group (20.6%, *p* = 0.02). Coronary artery disease was also more frequent in the non-mitral group (17.5%) than in the MR group (7.1%, *p* = 0.04).

Regarding operative data, 100% of cases in the MS group underwent mitral valve replacement (MVR), whereas 86.7% in the MR group primarily underwent mitral valve repair. Tricuspid annuloplasty (TAP) was performed more frequently in the MS (58.8%) and MR (61.6%) groups than in the non-mitral group (22.2%, *p* < 0.001). Concomitant CABG and ASD repair were performed significantly more frequently in the non-mitral group than in the MS and MR groups.

Regarding preoperative echocardiographic parameters, there were significant hemodynamic differences among the groups. The MR group showed the largest LVEDD (5.5 ± 0.7 cm), which was significantly greater than that in the MS (4.8 ± 0.5 cm, *p* < 0.001) and non-mitral (4.9 ± 0.7 cm, *p* < 0.001) groups. LAVI significantly increased in both the MS (110.7 ± 33.5 mL/m^2^) and MR (116.1 ± 48.5 mL/m^2^) groups compared with that in the non-mitral group (68.4 ± 20.1 mL/m^2^, *p* < 0.001 for both). However, the MS and MR groups showed no statistically significant difference in LAVI (*p* = 0.43). LVEF was preserved and comparable across all three groups.

Concerning AF characteristics, the proportion of persistent AF and the presence of fine f-waves were similar across all groups without statistical significance.

### 3.2. Changes in LASct

**Figure 2:**
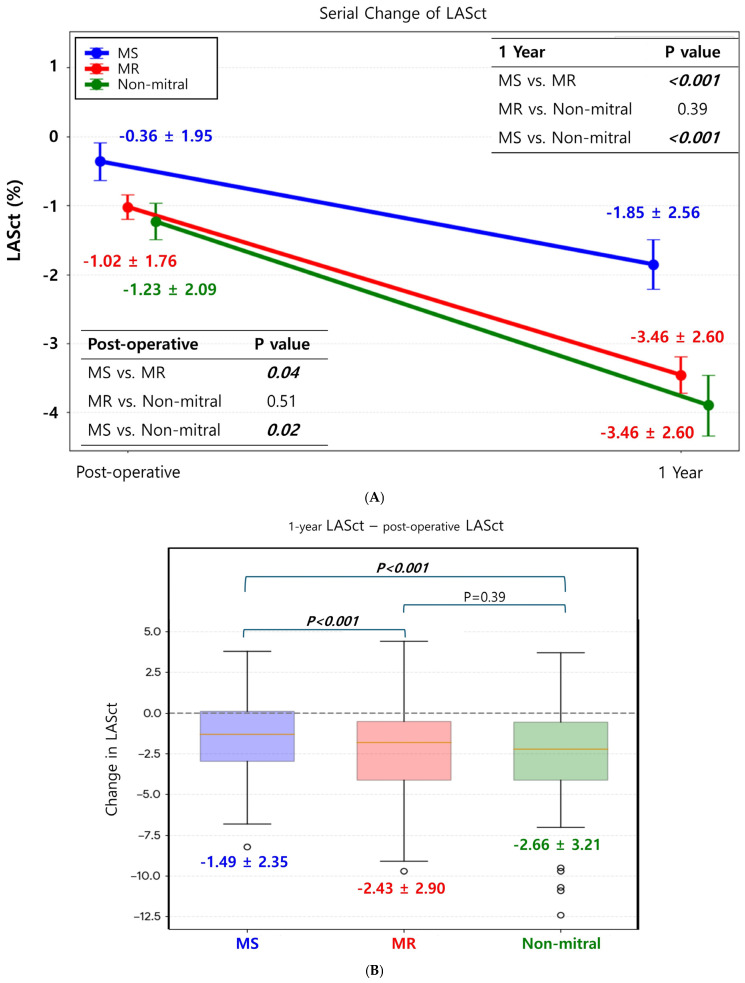
Serial changes in left atrial contractile (LASct) strain after surgery. (**A**) Improvement in LASct (becoming more negative) was observed in all three groups during the 1-year maintenance of normal sinus rhythm after the Maze procedure. (**B**) However, the magnitude of LASct improvement (defined as the difference between 1-year LASct and postoperative LASct) was significantly more prominent in the MR and non-mitral groups than in the MS group (*p* < 0.001 for both MR vs. MS and non-mitral vs. MS). In contrast, there was no significant difference in the degree of LASct improvement during the first postoperative year between the MR and non-mitral groups.

The immediate postoperative LASct values were −0.36 ± 1.95 in the MS group, −1.02 ± 1.76 in the MR group, and −1.23 ± 2.09 in the non-mitral group (*p* = 0.04 for MS vs. MR; *p* = 0.51 for MR vs. non-mitral; *p* = 0.02 for MS vs. non-mitral) (Figure 2A). At 1-year follow-up, the LASct improved to −1.85 ± 2.56 in the MS group, −3.46 ± 2.60 in the MR group, and −3.46 ± 2.60 in the non-mitral group (*p* < 0.001 for MS vs. MR; *p* = 0.39 for MR vs. non-mitral; *p* < 0.001 for MS vs. non-mitral). Regarding the change from the immediate postoperative period to 1 year (1-year LASct minus postoperative LASct) (Figure 2B), the mean reduction was −1.49 ± 2.35 in the MS group, −2.43 ± 2.90 in the MR group, and −2.66 ± 3.21 in the non-mitral group. Both the MR and non-mitral groups showed a significantly greater magnitude of LASct reduction than the MS group (*p* < 0.001 for both). The extent of LASct reduction was not significantly different between the MR and non-mitral groups (*p* = 0.39).

After adjusting for confounding factors using multivariable linear regression analysis to determine the impact of mitral valvular etiology on LA systolic function recovery (Table 2), the MR group demonstrated a significantly greater reduction in both the 1-year LASct value and the absolute change in LASct than the MS group (LASct at 1 year: coefficient −1.414, *p* = 0.03, 95% CI [−2.690, −0.138]; absolute change in LASct: coefficient −1.047, *p* = 0.03, 95% CI [−2.022, −0.073]). Conversely, the effect sizes for the differences between the non-mitral reference group and the MR group were small and not statistically significant, with confidence intervals crossing zero for both the 1-year LASct (coefficient 0.682, 95% CI [−0.381, 1.745], *p* = 0.21) and the absolute change in LASct (coefficient 0.624, 95% CI [−0.417, 1.665], *p* = 0.24).

### 3.3. Serial Changes in LA Reservoir and Conduit Strains

Table 3 summarizes the serial changes in LA reservoir (LASr) and LA conduit (LAScd) strains from the immediate postoperative period to 1 year. In the immediate postoperative period, there were significant differences in LA function among the groups. The non-mitral group showed the most preserved LA function, followed by the MR group, whereas the MS group exhibited the most impaired values. In particular, the MS group showed significantly lower LASr and less negative LAScd than the MR (*p* = 0.004 for LASr; *p* = 0.03 for LAScd) and non-mitral (*p* < 0.001 for both) groups.

At the 1-year follow-up, all groups demonstrated improvement in strain parameters. The hierarchical pattern of LA function (non-mitral > MR > MS) persisted for LASr, with significant differences observed among all groups. For LAScd, although the non-mitral group continued to exhibit significantly better function than the MS and MR groups (*p* < 0.001 for both), the difference between the MS and MR groups was statistically nonsignificant (*p* = 0.37).

Concerning functional recovery during the first postoperative year, represented by the absolute change (Δ) in strain values, all three groups exhibited comparable degrees of improvement. No statistically significant differences were observed in the magnitude of change for either Δ LASr (*p* > 0.05 for all comparisons) or Δ LAScd (*p* > 0.05 for all comparisons) among the three groups. However, it is important to note that the absolute magnitude of this improvement was clinically modest across all cohorts. This indicates a comparable, albeit limited, recovery of atrial compliance regardless of the underlying etiology.

### 3.4. Changes in Other Echocardiographic Parameters

**Figure 3:**
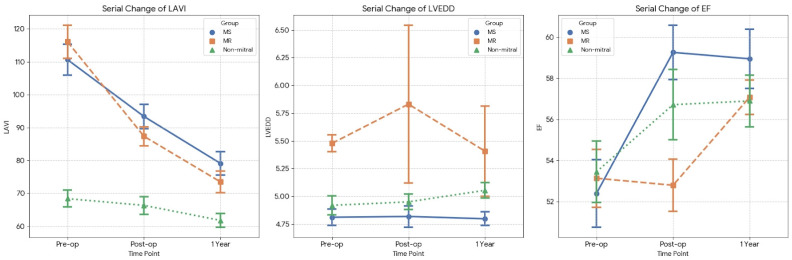
Serial changes in transthoracic echocardiographic parameters at preoperative, postoperative, and 1-year follow-up time points. **Left atrial volume index (LAVI):** LAVI decreased in all three groups. The extent of reduction was more prominent in the MS and MR groups than in the non-mitral group. **Left ventricular end-diastolic dimension (LVEDD):** There were no marked changes in LVEDD across the three groups when comparing preoperative and postoperative values. The MR group exhibited a transient increase in LVEDD immediately after surgery, which gradually returned to baseline levels over the 1-year follow-up. **Ejection fraction (EF):** In the MS and non-mitral groups, EF increased immediately after surgery and remained stable thereafter. In contrast, the MR group showed no immediate change in EF postoperatively but exhibited a gradual improvement over the 1-year period. Although the overall magnitude of EF improvement was similar across all three groups, the rate of recovery was slower in the MR group.

The serial changes in transthoracic echocardiographic parameters at preoperative, immediate postoperative, and 1-year follow-up time points are depicted in Figure 3. LAVI exhibited a decreasing trend in all three groups (Figure 3). The MR group showed a significantly greater reduction than both the MS and non-mitral groups. The MS group also exhibited a significant reduction in LAVI compared with the non-mitral group (Table 4).

Regarding LVEDD (Figure 3), the MR group exhibited a decrease at 1 year, whereas the MS group showed no significant change, and the non-mitral group demonstrated an increase. The reduction in LVEDD in the MR group was statistically significant compared with that in the other two groups (Table 4).

LVEF increased in all three groups at 1-year follow-up (Figure 3). However, although the MS and non-mitral groups exhibited improvement immediately after surgery, the MR group showed no significant immediate change, followed by delayed recovery. No significant difference was observed in the magnitude of EF change between the preoperative and 1-year time points among the three groups (Table 4).

### 3.5. MACE and PPM Implantation

**Figure 4:**
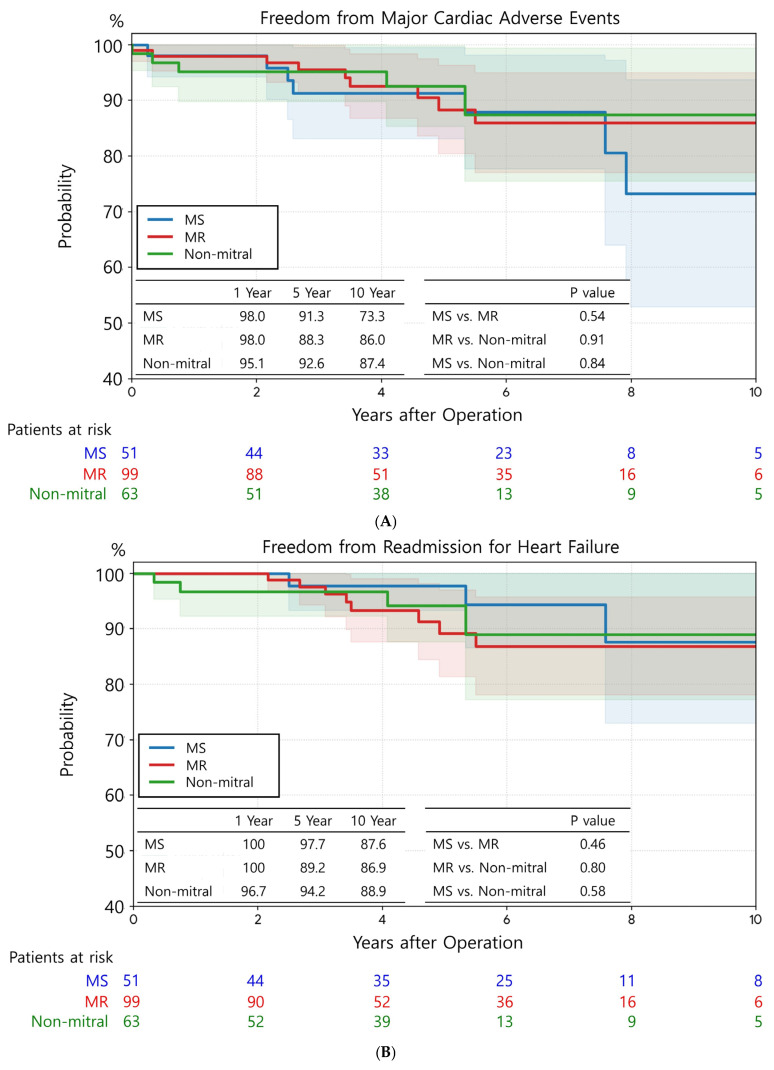

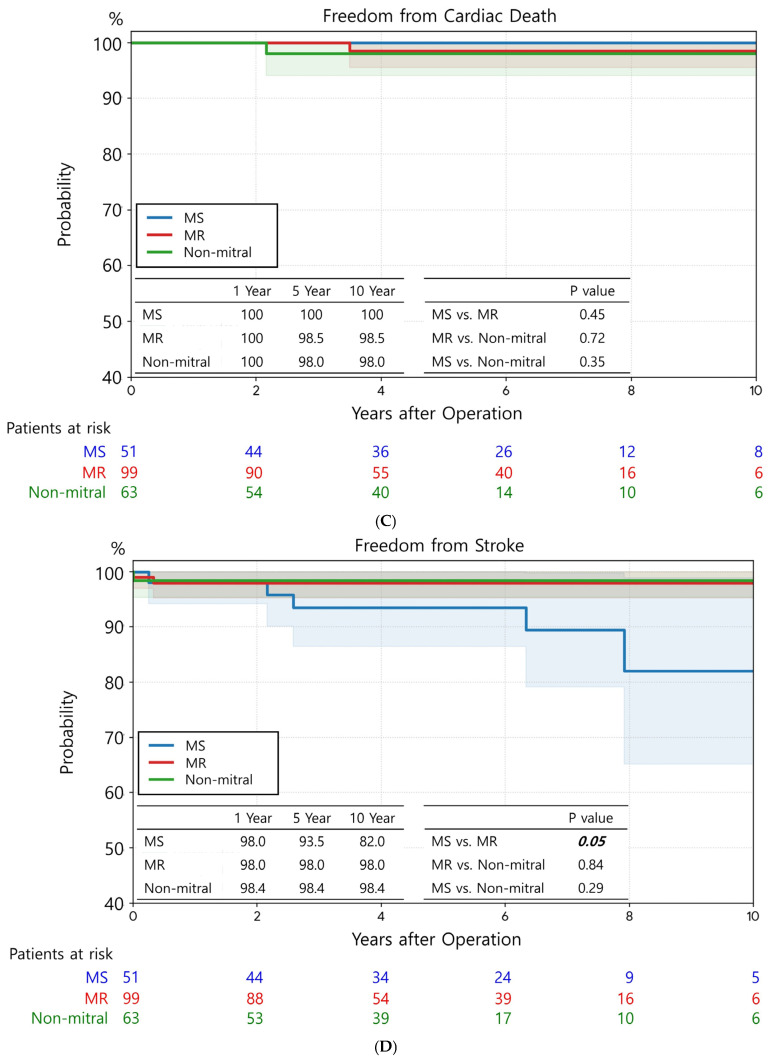
Clinical outcomes regarding major adverse cardiac Events (MACEs). There were no significant differences in MACE outcomes among the three groups. The shaded background represents the standard deviation. (**A**) Freedom from MACEs. (**B**) Freedom from readmission for heart failure. (**C**) Freedom from cardiac death. (**D**) Freedom from stroke.

The three groups showed no significant differences in freedom from MACEs (Figure 4A). The rates of freedom from MACEs at 1, 5, and 10 years were 98.0%, 91.3%, and 73.3% in the MS group; 98.0%, 88.3%, and 86.0% in the MR group; and 95.1%, 92.6%, and 87.4% in the non-mitral group, respectively (*p* = 0.54 for MS vs. MR; *p* = 0.91 for MR vs. non-mitral; *p* = 0.84 for MS vs. non-mitral). Similarly, no significant differences were observed among the groups in freedom from readmission for HF (Figure 4B) and cardiac death (Figure 4C).

Nonetheless, the MS group exhibited a significantly lower rate of freedom from stroke than the MR group (*p* = 0.05) (Figure 4D). Comparisons between MS vs. non-mitral (*p* = 0.29) and MR vs. non-mitral (*p* = 0.84) groups yielded no significant differences.

**Figure 5:**
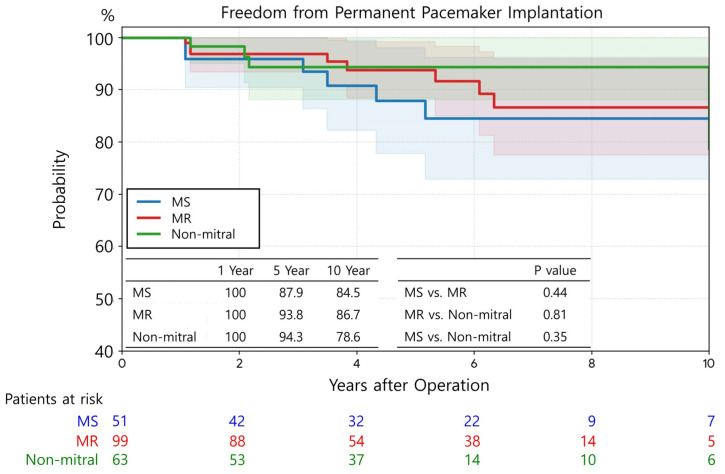
Multivariable Cox regression analysis for risk factors associated with stroke. The shaded background represents the standard deviation.

No significant predictors were identified in the risk factor analysis for stroke (Figure 5). The multivariable analysis also confirmed that MS was not a significant independent risk factor for stroke.

**Figure 6:**
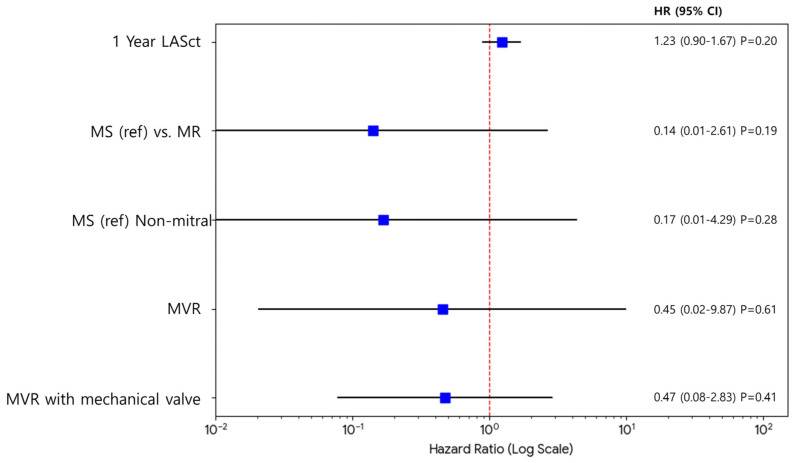
Freedom from permanent pacemaker implantation.

Freedom from PPM implantation was not significantly different among the three groups (Figure 6). The 5- and 10-year rates of freedom from PPM implantation were 87.9% and 84.5% in the MS group, 93.8% and 86.7% in the MR group, and 94.3% and 78.6% in the non-mitral group, respectively (*p* = 0.44 for MS vs. MR; *p* = 0.81 for MR vs. on-mitral; *p* = 0.35 for MS vs. non-mitral).

## 4. Discussion

### 4.1. Principal Findings

This study demonstrated that post-Maze functional recovery varies according to mitral etiology. First, despite restored sinus rhythm, LA mechanical recovery (LASct) was significantly impaired in the MS group, whereas the MR group achieved favorable reverse remodeling comparable with the non-mitral group. Second, the MR group showed a distinct “delayed” LV recovery pattern—transient stagnation followed by gradual improvement—in contrast to the immediate LVEF improvement observed in the MS and non-mitral groups.

### 4.2. Differential LA Functional Recovery: MS vs. MR

Although the Maze procedure successfully restored and maintained NSR across all the study groups, our results demonstrate that electrical recovery does not guarantee uniform functional recovery. In particular, despite the maintenance of sinus rhythm, the extent of LA mechanical recovery differed significantly depending on the etiology.

Although the 1-year change in LAScd was comparable between the MS and MR groups, the improvement in LASct was significantly more prominent in the MR group. These data indicate that the favorable LA reverse remodeling observed in patients with MR after the Maze procedure and mitral valve surgery is primarily driven by the restoration of active mechanical contraction (“atrial kicking”), rather than merely by improvements in LA compliance. In general, patients with MS exhibit persistent LA dysfunction, emphasizing the irreversible nature of chronic pressure overload-induced remodeling; however, patients with MR exhibit significantly greater improvement in LA function than those with MS [6,8]. This suggests that the volume-overloaded LA in MR retains contractile reserve using the Frank–Starling mechanism to regain pump function once the regurgitant volume is eliminated [8]. In stark contrast, the pressure overload in MS is hypothesized to lead to irreversible ultrastructural changes in the atrial myocardium, such as extensive interstitial fibrosis and myofiber atrophy, resulting in intrinsic myocardial failure [8,15,16,17]. Consequently, the MS group failed to regain effective contraction comparable to that in the MR group, despite similar improvements in stiffness. This emphasizes that for patients with MS, the restoration of electrical rhythm is a necessary but insufficient condition for functional recovery, as the underlying myocardial damage is irreversible.

Despite the statistically significant improvements observed in the MR and MS group, the absolute LASct values at 1 year remained substantially lower than normal reference ranges for atrial contractile strain (typically 8–15%) [18]. We believe this persistent global atrial dysfunction is primarily attributable to the fact that the baseline LA volume index (LAVI) in our patients was already profoundly enlarged prior to surgery. In patients with such severe preoperative LA enlargement, the capacity for LA reverse remodeling is inherently limited [9]. Consequently, even when the Maze procedure successfully restores electrical sinus rhythm, these patients cannot regain completely normal LA mechanical function [19]. Furthermore, while the 1.0–1.5% absolute difference in LASct recovery between the MR and MS groups appears numerically modest, it represents a clinically meaningful divergence. Specifically, it suggests that patients with MR regain a perceptible degree of active atrial ‘kick,’ whereas those with MS effectively remain in a passive conduit state.

In light of these findings, the persistent impairment of LASct in the MS group suggests potentially permanent structural alterations in the atrial myocardium, although direct histological confirmation was not performed in this study. This subclinical mechanical dysfunction—often termed ‘atrial myopathy’—raises important clinical considerations regarding long-term management. While current guidelines for post-Maze anticoagulation primarily rely on the restoration of sinus rhythm and clinical risk scores, our data imply that restored electrical rhythm does not necessarily equate to restored mechanical protection. Accumulating evidence suggests that impaired atrial mechanical function itself, independent of the rhythm status, is a potent substrate for thrombus formation [20]. Several studies have proposed that in patients with severe atrial remodeling and resultant ‘mechanical failure,’ continuing anticoagulation may be safer to prevent late thromboembolic events, even after the successful restoration of sinus rhythm [21]. Therefore, in patients with severely impaired LASct, a more cautious approach to anticoagulation management or intensified monitoring for subclinical arrhythmia might be warranted, as the absence of effective atrial ‘kick’ may predispose to blood stasis and subsequent stroke.

### 4.3. Delayed LV Functional Recovery in MR

Another remarkable finding in this study is the trajectory of LV systolic function recovery in the MR group. Although the MS and non-mitral groups exhibited immediate improvement in LVEF postoperatively, the MR group showed a delayed recovery pattern. This phenomenon can be explained by the alleviation of the “pop-off” effect and the unmasking of latent LV dysfunction [22,23]. In chronic MR, the low-impedance regurgitant pathway into the LA allows the LV to eject blood easily, often masking underlying myocardial dysfunction and maintaining the preoperative LVEF artificially preserved or elevated [23]. After mitral valve repair or replacement, this low-resistance pathway is eliminated, causing an immediate increase in LV afterload. Consequently, the ventricle faces an abrupt hemodynamic mismatch, resulting in a temporary stagnation or slight decrease in ejection fraction in the immediate postoperative period [22]. Nevertheless, our serial follow-up data demonstrated that this is a transient phenomenon; as the LV undergoes beneficial reverse remodeling—evidenced by the delayed recovery in LVEF and recovery of geometry—systolic function progressively improves over 1 year. Clinicians must be aware of this physiological lag in patients with MR to avoid misinterpreting the lack of immediate LVEF improvement as a procedural failure [24]. Importantly, despite these distinct early postoperative trajectories, the long-term LVEF at 1 year did not differ significantly among the three groups. This highlights that while the LV recovery patterns provide valuable pathophysiological context, they remain secondary observations; the primary and most enduring functional divergence determined by valvular etiology lies within the LASct.

### 4.4. Limitations

This study has several limitations. First, excluding patients with recurrent AF precludes evaluating the impact of persistent arrhythmia on reverse remodeling. Second, substantial surgical heterogeneity existed; all MS patients underwent valve replacement (MVR), whereas 86.7% of MR patients underwent repair. Because MVR disrupts annuloventricular continuity, the surgical technique itself may independently hinder mechanical recovery, potentially confounding the impaired LASct observed in the MS group. Third, our mechanistic hypotheses regarding irreversible fibrosis lack direct histological or advanced imaging validation (e.g., MRI T1 mapping), making our pathophysiological interpretations speculative. Fourth, impaired LASct was not statistically associated with stroke risk (*p* = 0.20), likely due to our limited sample size and low event rates, requiring larger long-term studies for confirmation. Fifth, we did not perform formal inter-observer or intra-observer reliability testing for speckle-tracking echocardiography, which involves manual endocardial border tracing. Finally, the retrospective, non-randomized design limits causal inference, while the reliance on a single-center cohort restricts demographic diversity and generalizability. Despite multivariable adjustments, inherent selection bias and unmeasured confounders including lifestyle factors and detailed medication adherence cannot be entirely excluded.

## 5. Conclusions

In conclusion, the restoration of electrical sinus rhythm through the Maze procedure does not guarantee uniform recovery of left atrial mechanical function across different mitral etiologies. Patients with MS exhibit significantly impaired LA contractile recovery compared to those with MR or non-mitral etiologies, suggesting that persistent subclinical atrial myopathy may persist despite successful electrical conversion. While these functional discrepancies are hypothesized to stem from irreversible structural remodeling in MS, our findings highlight that electrical success should not be equated with functional restoration. Clinically, these results suggest that a more individualized approach to postoperative management may be warranted. Specifically, in patients with severely impaired LASct, clinicians should exercise caution when considering the discontinuation of anticoagulation and consider intensified monitoring for subclinical thromboembolic events, regardless of the apparent maintenance of sinus rhythm.

## Figures and Tables

**Table 1:** Patients’ characteristics.

Variable	MS (N = 51)	MR (N = 98)	Non-Mitral (N = 62)	*p* Value		
				MS vs. MR	MR vs. Non-Mitral	MS vs. Non-Mitral
Age, year	60.5 ± 9.8	63.5 ± 9.6	63.4 ± 7.8	0.07	0.89	0.09
Sex (male) (%)	16 (31.4)	45 (45.5%)	38 (60.3)	0.14	0.09	***0.004***
**Comorbidities (%)**						
Diabetes	9 (17.6)	12 (12.1)	16 (25.4)	0.50	***0.05***	0.44
Coronary disease	2 (3.9)	7 (7.1)	11 (17.5)	0.72	0.07	***0.04***
Stroke	9 (17.6)	8 (8.1)	16 (25.4)	0.14	***0.005***	0.44
Heart failure	15 (29.4)	39 (39.4)	13 (20.6)	0.30	***0.02***	0.39
COPD	3 (5.9)	6 (6.1)	1 (1.6)	>0.99	0.25	0.32
CKD	0 (0.0)	5 (5.1)	2 (3.2)	0.17	0.71	0.50
CKD on dialysis	0 (0.0)	0 (0.0)	1 (1.6)	>0.99	0.39	>0.99
Redo-sternotomy	3 (5.9)	7 (7.1)	1 (1.6)	>0.99	0.15	0.32
**Concomitant procedures**						
TAP	30 (58.8)	61 (61.6)	14 (22.2)	0.88	***<0.001***	***<0.001***
CABG	2 (3.9)	5 (5.1)	13 (20.6)	>0.99	***0.005***	***0.01***
ASD	2 (3.9)	7 (7.1)	18 (28.6)	0.72	***<0.001***	***<0.001***
Myxoma	0 (0.0)	0 (0.0)	2 (3.2)	>0.99	0.15	0.50
MVR	51 (100)	11 (13.3)	0	***<0.001***	***0.007***	***<0.001***
Mitral valve repair	0	88 (86.7)	0	***<0.001***	***<0.001***	>0.99
**Atrial fibrillation (AF) details**						
Persistent AF	39 (76.5)	79 (79.8)	48 (76.2)	0.79	0.73	>0.99
Fine F-wave	24 (47.1)	52 (52.5)	33 (52.4)	0.64	>0.99	0.71
**Preoperative echocardiography results**						
LVEDD	4.8 ± 0.5	5.5 ± 0.7	4.9 ± 0.7	***<0.001***	***<0.001***	0.35
Ejection fraction	52.4 ± 11.7	53.1 ± 13.9	53.4 ± 11.9	0.73	0.88	0.64
LAVI	110.7 ± 33.5	116.1 ± 48.5	68.4 ± 20.1	0.43	***<0.001***	***<0.001***

Significant *p* values are depicted in italics and bolds. COPD, chronic obstructive pulmonary disease; CKD, chronic kidney disease; TAP, tricuspid annuloplasty; CABG, coronary artery bypass graft; ASD, atrial septal defect; MVR, mitral valve replacement; LVEDD, left ventricular end-diastolic dimension; LAVI, left atrial volume index.

**Table 2:** Adjusted comparison of LASct changes at 1-year follow-up among the three groups (confounding factors were adjusted for using multivariable linear regression analysis).

Comparison	Coefficient	*p* Value	95% CI
**^(a)^ LASct at 1 year after operation**			
MR (Ref.) vs. MS	−1.414	***0.03***	[−2.690, −0.138]
Non-mitral (Ref.) vs. MS	−0.732	0.37	[−2.345, 0.882]
Non-mitral (Ref.) vs. MR	0.682	0.21	[−0.381, 1.745]
**^(b)^ Absolute change in LASct during 1 year after operation**			
MR (Ref.) vs. MS	−1.047	***0.03***	[−2.022, −0.073]
Non-mitral (Ref.) vs. MS	−0.423	0.47	[−1.569, 0.723]
Non-mitral (Ref.) vs. MR	0.624	0.24	[−0.417, 1.665]

Significant *p* values are shown in italics and bolds. MR, mitral regurgitation; MS, mitral stenosis. ^(a)^ Adjustment variable: Baseline A-fib type, Preoperative LAVI, Mitral valve replacement. ^(b)^ **1-yea LASct—postoperative LASct**, Adjustment variable: Preoperative LAVI, Mitral valve replacement.

**Table 3:** Changes in left atrial reservoir and conduit (LASr and LAScd) strains from baseline to 1 year.

	Variable	MS (N = 51)	MR (N = 98)	Non-Mitral (N = 62)	*p* Value		
					MS vs. MR	MR vs. Non-Mitral	MS vs. Non-Mitral
**Postop**	LASr (%)	8.17 ± 3.89	10.14 ± 3.78	12.29 ± 6.43	***0.004***	***0.02***	***<0.001***
	LAScd (%)	−7.33 ± 3.22	−8.64 ± 3.83	−10.46 ± 5.63	***0.03***	***0.03***	***<0.001***
**At 1 Year**	LASr (%)	10.95 ± 4.50	12.88 ± 4.83	16.33 ± 7.06	***0.017***	***<0.001***	***<0.001***
	LAScd (%)	−9.10 ± 4.15	−9.73 ± 3.96	−12.77 ± 5.04	***0.37***	***<0.001***	***<0.001***
***1 Year post***	Δ LASr	+2.78 ± 5.23	+2.74 ± 5.51	+4.04 ± 6.53	0.97	0.19	0.25
	Δ LAScd	−1.77 ± 4.93	−1.09 ± 4.72	−2.31 ± 5.76	0.42	0.16	0.59

Significant *p* values are shown in italics and bolds.

**Table 4:** Serial changes in echocardiographic parameters.

Variable	MS (N = 51)	MR (N = 98)	Non-Mitral (N = 62)	*p* Value		
				MS vs. MR	MR vs. Non-Mitral	MS vs. Non-Mitral
**From the preoperative period to 1-year follow-up**						
^(a)^ LAVI change (%)	−24.9 ± 24.6	−34.3 ± 20.0	−5.3 ± 24.9	***0.02***	***<0.001***	***<0.001***
^(b)^ LVEDD change (%)	0.3 ± 9.8	−8.0 ± 10.4	3.9 ± 13.0	***<0.001***	***<0.001***	0.10
^(c)^ EF change (Abs)	6.6 ± 11.6	4.0 ± 12.2	3.4 ± 8.1	0.21	0.73	0.11
**From the immediate postoperative period to 1-year follow-up**						
^(d)^ LAVI change (%)	−24.9 ± 24.6	−34.3 ± 20.0	−5.3 ± 24.9	***0.02***	***<0.001***	***<0.001***
^(e)^ LVEDD change (%)	1.0 ± 12.8	−2.3 ± 10.0	2.4 ± 9.7	0.12	***0.004***	0.50
^(f)^ EF change (Abs)	−0.3 ± 8.5	4.3 ± 10.7	−0.1 ± 10.2	***0.005***	***0.01***	0.89

Significant *p* values are depicted in italics and bolds. LAVI, left atrial volume index; LVEDD, left ventricular end-diastolic dimension; EF, ejection fraction. ^(a)^ (1-year LAVI—preoperative LAVI)/preoperative LAVI × 100. ^(b)^ (1-year LVEDD—preoperative LVEDD)/preoperative LVEDD × 100. ^(c)^ 1-year EF—preoperative EF. ^(d)^ (1-year LAVI—postoperative LAVI)/postoperative LAVI × 100. ^(e)^ (1-year LVEDD—postoperative LVEDD)/postoperative LVEDD × 100. ^(f)^ 1-year EF—postoperative EF.

## Data Availability

The data presented in this study are available on request from the corresponding author. The data are not publicly available due to privacy and ethical restrictions.

## Abbreviations

The following abbreviations are used in this manuscript:

AF	Atrial fibrillation
NSR	Normal sinus rhythm
LA	Left atrium
LASct	Left atrial contractile
MS	Mitral stenosis
MR	Mitral regurgitation
TTE	Transthoracic echocardiography
LVEF	Left ventricular ejection fraction
LAVI	Left atrial volume index
LVEDD	Left ventricular end-diastolic dimension
MACE	Major adverse cardiac event
RA	Right atrium
HF	Heart failure
